# Broadband Zero-Power Wakeup MEMS Device for Energy-Efficient Sensor Nodes

**DOI:** 10.3390/mi13030407

**Published:** 2022-03-02

**Authors:** Minhaz Ahmed, Torben Dankwort, Sven Grünzig, Volker Lange, Björn Gojdka

**Affiliations:** 1 Fraunhofer Institute for Silicon Technology ISIT, Fraunhoferstr. 1, 25524 Itzehoe, Germany; minhaz.ahmed@isit.fraunhofer.de (M.A.); torben.dankwort@isit.fraunhofer.de (T.D.); sven.gruenzig@isit.fraunhofer.de (S.G.); 2 Mechanical and Medical Engineering, Hochschule Furtwangen University, Robert-Gerwig-Platz 1, 78120 Furtwangen im Schwarzwald, Germany; volker.lange@hs-furtwangen.de

**Keywords:** zero-power wakeup, lossless standby, piezoelectric MEMS energy harvesting, wafer-level-integrated micromagnets, wireless sensor nodes WSN, GreenICT, energy efficiency

## Abstract

A zero-power wakeup scheme for energy-efficient sensor applications is presented in this study based on a piezoelectric MEMS energy harvester featuring wafer-level-integrated micromagnets. The proposed setup overcomes a hybrid assembly of magnets on a chip-level, a major drawback of similar existing solutions. The wakeup device can be excited at low frequencies by frequency up-conversion, both in mechanical contact and contactless methods due to magnetic force coupling, allowing various application scenarios. In a discrete circuit, a wakeup within 30–50 ms is realized in frequency up-conversion at excitation frequencies < 50 Hz. A power loss in the off state of 0.1 nW renders the scheme virtually lossless. The potential extension of battery lifetime compared to cyclical wakeup schemes is discussed for a typical wireless sensor node configuration.

## 1. Introduction

Today, billions of sensors form the interface between the IoT and its physical environment [1]. Due to the ongoing digitalization and numerous applications based on the IoT, the number of sensor nodes is growing at a high pace [2]. In the face of the sheer amount of IoT entities, power consumption has become increasingly important from an environmental point of view. In addition, a power supply, e.g., a battery, might have to meet a minimum lifetime requirement to make the use of sensors in certain applications economically viable. This is the case especially in application scenarios where the deployment and regular replacement of a sensor node is cumbersome. Besides energy harvesting for fully self-powered devices [3,4,5], numerous concepts have been proposed on software and hardware levels to increase the lifetime of autonomous sensor nodes by a reduction in unwanted energy loss [6,7]. Since the power consumption in standby between measurement events can noticeably shorten the battery life of a sensor node, a major direction of development aims at the realization of zero-power standby solutions to extend battery lifetime [6].

A common zero-power standby scheme utilizes a transistor switch to cut off the power supply of a device when it does not needed to operate [6]. In this case, a wakeup unit is required which provides a voltage signal at the transistor gate at specific wakeup events. Various wakeup units have been proposed, such as devices responding to optical [6], RF [8], mechanical [9], and combined [7] excitation signals, or utilizing the triboelectric effect [10,11]. It depends on the requirements of a specific application which of these approaches is best suited. Parameters to be considered include the available sources of environmental energy in the application, the required wakeup time, and constraints regarding size and costs. Piezoelectric MEMS energy harvesters target mechanical energy sources. They offer a high level of miniaturization and a high power density [12] which can provide short wakeup times. Accordingly, numerous piezoelectric MEMS devices for (near) zero-power wakeup have been presented, e.g., [7,13,14,15].

**Figure 1:**
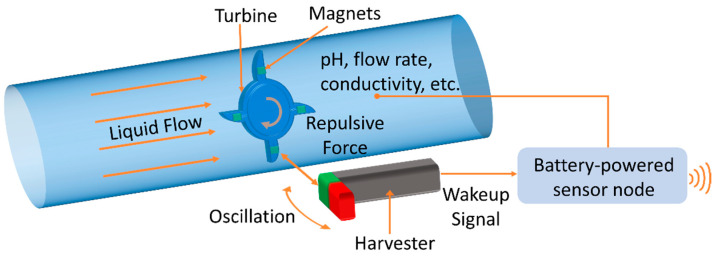
Application scenario of a magnetic MEMS-based wakeup device for a water monitoring system. A low-frequency rotary motion triggers the wakeup of a battery-powered WSN in a contactless way.

In general, MEMS technology is potentially suitable for the cost-efficient fabrication of miniaturized and integrated wakeup units. However, typical resonance frequencies of vibrating MEMS structures are commonly used in the kHz regime, making them unsuitable for the detection of low-frequency wakeup events. In addition, the frequency response of such devices is usually narrow, adding the necessity to adapt the device design to a specific use case. Furthermore, depending on the application scenario, contactless excitation might be required. An example for an application requiring contactless low-frequency excitation and a short wakeup time is depicted in Figure 1. In this scenario, the battery lifetime of a WSN of a water monitoring system is extended by zero-power standby. A flow of some medium in the pipe triggers the wakeup without contact due to the low-frequency rotary motion of the turbine. Such a system can be utilized, for example, for smart water monitoring, which is of fundamental relevance in the face of the increasing scarcity of fresh water [16,17].

Various techniques have been proposed to extent the usable frequency range, such as frequency up-conversion, non-linear excitation, and resonance tuning [18,19]. These concepts have been realized in several devices by making use of magnetic forces [18]. However, due to the limitations of established MEMS fabrication processes, the permanent magnetic components in these devices are hybrid assembled, which renders fabrication cumbersome and limits miniaturization. A novel fabrication technique [20] enables the integration of three-dimensional hard magnetic structures into MEMS on a wafer level [21]. Using this technique, a fully wafer-level-fabricated MEMS energy harvester with integrated permanent magnets was fabricated [22]. The device enables the utilization of broadband low-frequency mechanical and magnetic excitation, making it suitable for linear and rotational motions and operation with and without mechanical contact. A detailed theoretical and experimental study of the performance of the MEMS energy-harvesting device can be found in [22].

Based on this MEMS device, a contactless low-frequency zero-power standby is demonstrated in this work. The power loss during standby is determined and the potential increase in the battery lifetime of an autonomously powered sensor node is compared to a fixed-cycle wakeup scheme. To the best of our knowledge, this is the first fully wafer-level-fabricated MEMS device with integrated permanent magnets used for zero-power wakeup.

## 2. Theory

Typical resonance frequencies of MEMS devices are in the kHz regime. To address low-frequency wakeup events, the frequency up-conversion technique is used. In this scheme, an external low-frequency event with an excitation frequency f_exc_  ≪ f_res_ well below the resonance frequency f_res_ is utilized to excite the harvester. In between the excitations, the cantilever oscillates at its resonance frequency.

**Figure 2:**
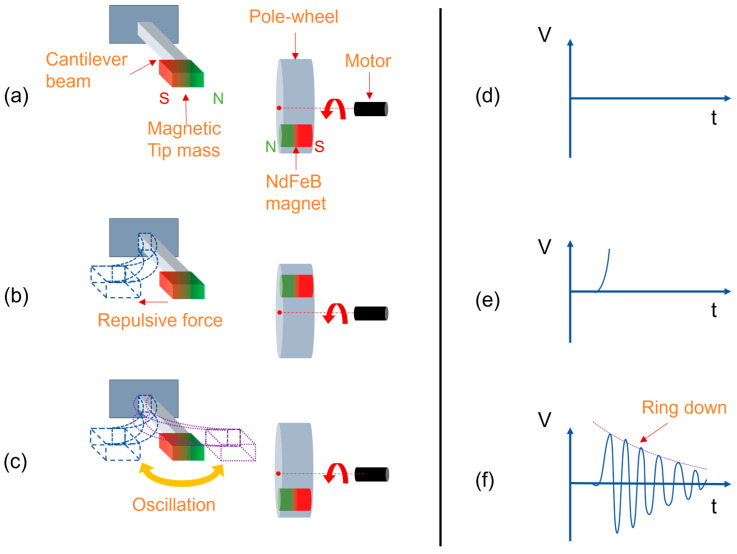
Illustration of the magnetic plucking-based frequency up-conversion scheme. (**a**) Pole-wheel magnet is far away before plucking, (**b**) magnet is at the closest distance to the tip mass during plucking, (**c**) the excitation magnet moves away from the harvester after plucking. (**d**–**f**) illustrate the voltage output of the harvester at the respective stages of plucking.

The realization of frequency up-conversion schemes can be classified into contact based, e.g., [9,23,24,25], and contactless methods, e.g., [26,27,28,29]. The contact-based schemes are characterized by introducing mechanical impact to the harvester. In contrast, force coupling in the contactless methods occurs between magnets placed at an oscillator and external magnets. In this work, contactless magnetic plucking was realized by a rotating wheel incorporating a magnet which periodically approaches the MEMS wakeup device, as illustrated in Figure 2. The generated repulsive force induces a deflection of the cantilever, leaving it to oscillate at its eigenfrequency upon removal of the external magnetic force. The complex excitation mechanism of magnetic plucking has been investigated in-depth elsewhere [28,29,30].

Due to the underdamped oscillation of the cantilever, the amplitudes of the voltage response as illustrated in Figure 2f follow an exponentially decaying envelope. Several works have focused on formulating a dynamic analysis of piezoelectric beams under vibration-based excitations [31,32] and magnetic plucking [28,29,30,33,34]. An intrinsic feature of the frequency up-conversion scheme is the periodic increase and decrease in the oscillator’s voltage output upon sweeping f_exc_ [29,34,35]. This phenomenon occurs due to in- or out-of-phase excitation of the still oscillating cantilever [29]. Accordingly, the corresponding excitation frequencies of output voltage maxima and minima can be determined by the relationships

(1) $$f_{n,\max}=\frac{f_{\mathrm{res}}}{n}\;\mathrm{and}\;f_{n,\min}=\frac{f_{\mathrm{res}}}{n+0.5}$$

## 3. Experimental Setup

**Figure 3:**
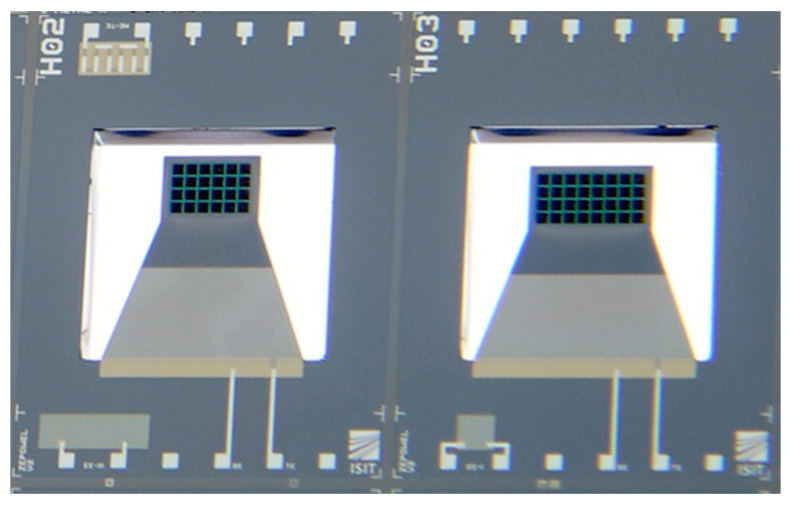
Photograph of the two MEMS designs used for the wakeup scheme. Micromagnets made of NdFeB powder are wafer-level integrated into the cantilever tips (black squares). Refer to Table 1 for dimensions.

The facilitated MEMS devices to transduce the mechanical or magnetically induced shock is based on a trapezoidal cantilever structure (29 µm poly-Si) coated with a piezoelectric aluminum nitride (AlN) film of 2 µm thickness. In fully integrated MEMS energy harvesters, a seismic mass made of silicon is typically used to tune the mechanical properties of the oscillator. However, a novel integration technology allows us to replace these silicon masses with materials of higher density and magnetic properties on a wafer level [36]. In this study, micromagnets made of microfine NdFeB powder (Magnequench MQFP-B+) were wafer-level integrated and subsequently magnetized perpendicular to the wafer surface at H = 2800 kA/m. The two MEMS designs used throughout this study are shown in Figure 3. Table 1 summarizes the major design parameters of the devices. For a more detailed discussion regarding the design aspects of the harvesters, readers are referred to [22,36].

**Figure 4:**
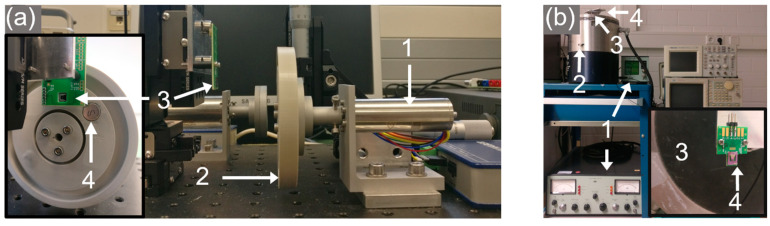
Excitation and measurement setups. (**a**) Pole-wheel setup: 1—motor, 2—pole-wheel, 3—harvester and 4—magnet. In the inset, the mounting of the harvester is shown from a different perspective. (**b**) Shaker setup: 1—driver electronics, 2—mechanical exciter body, 3—movable membrane, 4—harvester. The inset illustrates the mounting of the harvester from a top view.

The wakeup unit is excited by either contactless means, using a magnetic pole-wheel to mimic a rotational movement in Figure 4a, or in contact by a mechanical shaker set-up, as in Figure 4b. The pole-wheel setup houses two cylindrical NdFeB magnets stacked together (N52 grade, 10 mm height, and 5 mm diameter). The wheel is driven by a DC motor (Faulhaber 2264W012BP4) and the harvester is mounted on a three-axis stage kit to position it relative to the pole wheel (XRN25P-K2/M, Thorlabs).

The second setup excites the harvester without magnetic forces by acceleration in mechanical contact using a shaker setup. For this study, the integrated NdFeB structures of the MEMS devices were unmagnetized and the harvester was firmly attached onto a moving membrane. The mechanical excitation body (Brüel & Kjær, Darmstadt, Germany, Type 4805) is driven by a combination of a power amplifier (Brüel & Kjær, Darmstadt, Germany, Type 2707) and a charge amplifier (Brüel & Kjær, Darmstadt, Germany, Type 2525). For calibration and data acquisition, a DeltaTron reference acceleration sensor (Brüel & Kjær, Type 4396) and an oscilloscope was used (Tektronix, Cologne, Germany, MDO 3034). A waveform generator (Tektronix, Cologne, Germany, AWG2005) provided a customized excitation signal input to the power amplifier.

**Figure 5:**
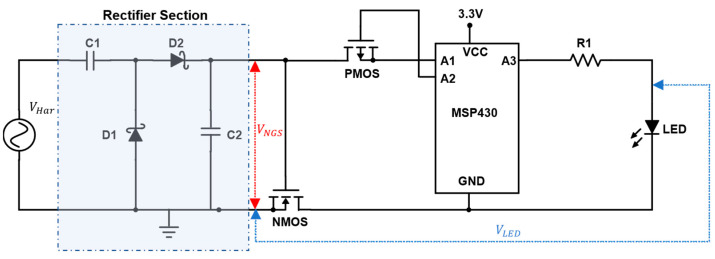
Wakeup circuitry schematic. Refer to text for details.

For the demonstration of a wakeup device, an NMOS transistor (TN0702, Supertex, maximum threshold voltage 1 V) acting as a footer switch was used in the wakeup circuit illustrated in Figure 5. A Greinacher voltage doubler circuit rectifies the harvester’s voltage output. Upon reaching the gate voltage required to switch the NMOS, a microcontroller (MSP430) is connected to a 3.3 V battery DC power supply. After waking up, the microcontroller drives the footer switch with a PMOS (LP0701, Microchip, maximum threshold voltage = −1 V), making the on-state independent of the wakeup unit for the time of execution. An LED is controlled to blink as a placeholder for a device to be woken up, e.g., a wireless sensor node. After executing the program, the microcontroller disconnects the PMOS, thereby disconnecting itself from GND. In this state, the standby power consumption of the microcontroller is limited to the leakage current of the NMOS transistor. The latter was measured using a precision semiconductor parameter analyzer (Hewlett Packard, Palo Alto, California, USA, HP4156A) to determine power loss in the off state.

## 4. Experimental Results and Discussion

The output voltage of the harvester was basically characterized in resonance for mechanical and contactless magnetic excitation. Subsequently, output in the case of frequency up-conversion was investigated for a wakeup scenario with rotary motion. A thorough theoretical and experimental discussion of the energy harvester output is given in [22]. After basic characterization, zero-power wakeup was demonstrated with the device. Finally, the power loss during standby was determined.

### 4.1. Magnetic Excitation

**Figure 6:**
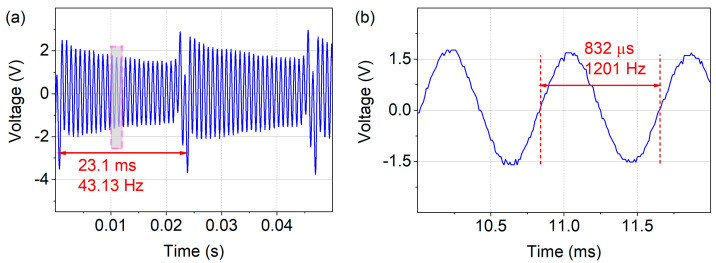
(**a**) Open circuit voltage output of V_PP_ ≈ 3–4 V in magnetic frequency up-conversion excitation at f_exc_ = 43.13 Hz. (**b**) The harvester oscillates at f_res_ = 1201 Hz in between the excitation events.

To characterize the voltage output in resonance in magnetic excitation, a pole-wheel (Figure 4a) was driven at 2600 RPM with a 2 mm separation distance between the harvester and the excitation magnet. Figure 6a depicts the voltage output for magnetic excitation at f_exc_ = 43.13 Hz. In between the magnetic excitation pulses, the harvester oscillated at its eigenfrequency f_res_ = 1201 Hz, Figure 6b. In this excitation setup, an open circuit voltage output of V_PP_ ≈ 3–4 V was provided by the harvester.

### 4.2. Mechanical Excitation

**Figure 7:**
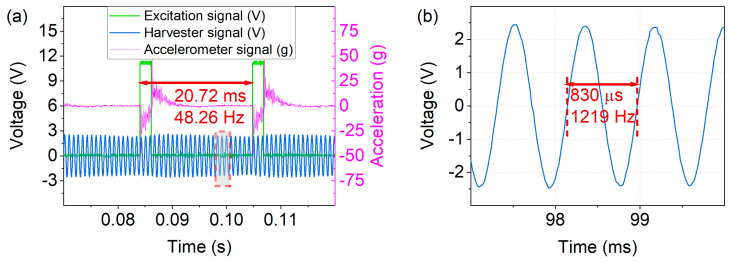
Mechanical excitation of unmagnetized devices. (**a**) A driving signal (f_exc_ = 48.26 Hz, 10% duty) results in acceleration peaks of 51 g. (**b**) In between the excitation pulses the harvester oscillates at it eigenfrequency f_res_ = 1219 Hz, delivering an output voltage of V_PP_ ≈ 5 V.

The voltage output of unmagnetized harvesters resulting from purely mechanical excitation was investigated in the shaker setup. A pulse signal with 10% duty cycle and f_exc_ = 30–50 Hz was used to drive the shaker resulting in an acceleration amplitude of a_exc,peak_ = 51 g, Figure 7a. An output of V_PP_ ≈ 5 V was generated in the frequency up-conversion at f_exc_ = 48.26 Hz, with a resonant oscillation at f_res_ = 1219 Hz in between the low-frequency excitation pulses, as shown in Figure 7b.

### 4.3. Broadband Low-Frequency Excitation

**Figure 8:**
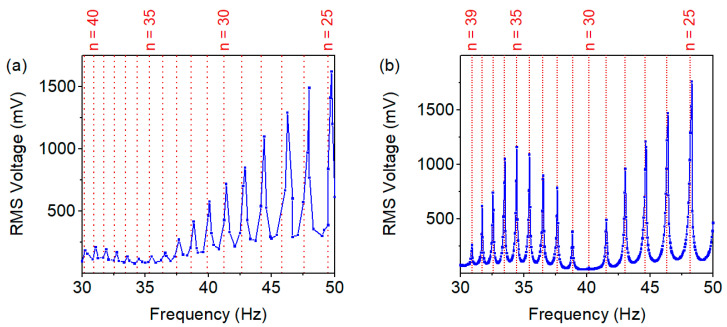
Voltage output across optimal load (R_opt_ ≈ 225 kΩ) in low-frequency up-conversion. (**a**) Contactless magnetic excitation. (**b**) In-contact mechanical excitation. The periodic extrema result from in- and out-of-phase excitation (see Equation (1) in Section 2).

For the wakeup device to respond to various events such as mechanical vibrations or slow rotational movements, broadband sensitivity is desirable, especially at low frequencies. Thus, the excitation frequency range f_exc_ = 30–50 Hz was studied in magnetic and mechanical excitation to demonstrate broadband harvesting capability. Accordingly, optimal loads for the specific harvester samples were applied by attaching a resistor decade box to the harvester and determining the load corresponding to maximum power output. The voltage response was investigated under a frequency sweep from 30–50 Hz. Both the magnetic (Figure 8a) and mechanical (Figure 8b) excitation schemes yielded the required wakeup voltage of V_gate_ ≥ 1 V at f_exc_ ≥ 40 Hz. Note that these outputs were setup-specific; in the presented measurements, a separation distance of d = 2 mm between harvester and excitation magnet was used in the contactless magnetic case, and for mechanical excitation an acceleration of a_exc_ = 50 g was employed. The periodic extrema in voltage output resulted from in- and out-of-phase excitation, as discussed in the theory section (Equation (1)). For comparison, the theoretically expected maxima are denoted in Figure 8, being in close agreement with the experimental data. As demonstrated, the frequency up-conversion scheme enabled the device to harvest at frequencies far below resonance.

### 4.4. Wakeup Demonstration

**Figure 9:**
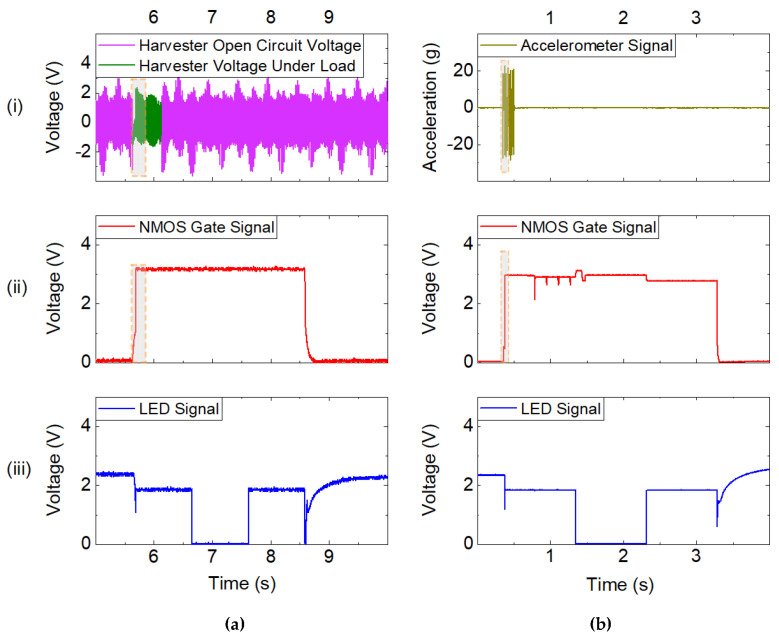
Wakeup of a microcontroller with subsequent program execution: (**a**) with contactless magnetic excitation of the harvester; (**b**) wakeup with mechanical excitation. The wakeup processes in the dashed sections are displayed in more detail in Figure 10.

For the demonstration of zero-power standby, a battery-powered microcontroller was woken up by the MEMS harvesting device. Figure 9 illustrates a whole wakeup, execution, and shutdown event of the microcontroller. The harvester is driven under open circuit conditions either magnetically, as depicted in Figure 9a, or mechanically, as shown in Figure 9b. Upon connection to the circuit, the harvester charges the capacitors used in the rectifier section. When the NMOS gate voltage reaches the gate threshold voltage of approximately 0.7 V, the NMOS connects the ground path of the microcontroller and thus wakes it up. The latter subsequently controls the driving of the NMOS, stabilizing the NMOS gate voltage at 3.3 V during the execution of the program task, as shown in Figure 9a,b (ii). From then on, for around 2.5 s the gate voltage of the NMOS is solely driven by the microcontroller, independent of the harvester signal. While this generic approach allows the wakeup of any device, an LED was made to flash for the purpose of demonstration. The proper execution is indicated by the periodic variation in LED voltage, as shown in Figure 9a,b (iii). Consequently, the supply to the NMOS gate voltage is cut off and the ground connection of the microcontroller floats again. Accordingly, the LED voltage returns to the open circuit voltage of that node, due to the floating ground connection.

**Figure 10:**
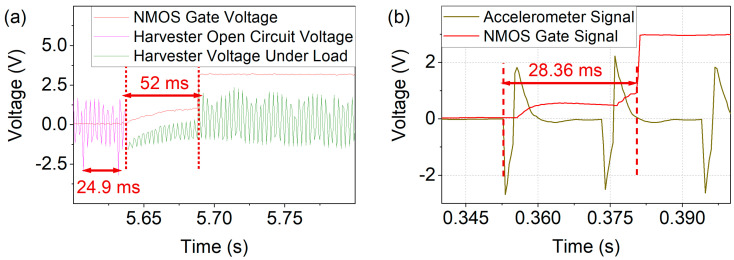
Time resolved wakeup process corresponding to the dashed intervals in Figure 9. (**a**) Voltage output of the harvester in magnetic excitation (f_exc_ = 40 Hz). Upon connection to the circuit the capacitors are charged accompanied by a drop in output voltage due the load. (**b**) Mechanical excitation in which the necessary gate voltage is reached after two acceleration pulses at f_exc_ = 48.14 Hz and a_exc_ = 22 g.

To determine the time and excitation required for wake up, the wakeup processes (dashed in Figure 9) were studied in more detail, as shown in Figure 10. As displayed in Figure 10a the voltage provided by the harvester dropped upon connection to the load of the wakeup circuit. When the capacitors were completely charged, the harvester’s voltage corresponded to the open circuit output. Upon magnetic excitation at a frequency of f_exc_ = 40 Hz, the gate voltage threshold V_gate_ ≥ 0.7 V was reached within 52 ms with C_1,2_ = 47 nF. Thus, two magnetic excitation pulses suffice until the NMOS controls V_gate_. The corresponding process, in the case of mechanical excitation, is displayed in Figure 10b. The time of 28.4 ms from the start of the excitation until V_gate_ is controlled by the microcontroller corresponding to two acceleration pulses f_exc_ = 48.14 Hz with C_1,2_ = 2.7 nF.

### 4.5. Stand-by Power Loss

**Figure 11:**
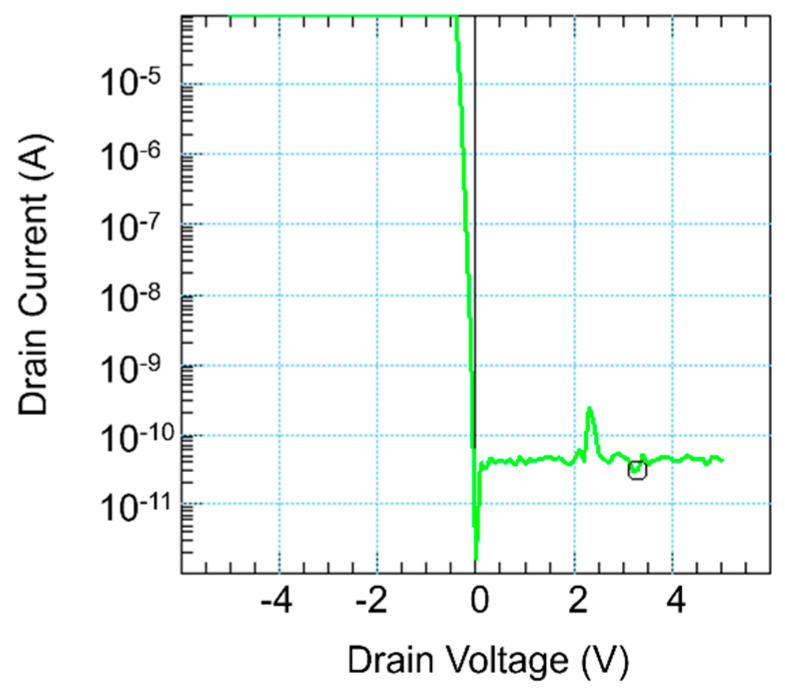
Measurement of the leakage current of the footer NMOS transistor used in the circuit shown in Figure 5.

The power loss in the off state is limited to the leakage current flowing through the NMOS transistor. To determine the leakage current, the drain voltage was swept from −5 V to 5 V in the off state of the NMOS and the corresponding drain current was measured, as shown in Figure 11. At a DC voltage of 3.3 V, which corresponds to the battery supply voltage of the microcontroller, the leakage current was 31.1 pA. Thus, the power loss during standby was 0.1 nW.

## 5. Estimation of Wakeup Benefit for Battery Lifetime Improvement

The presented device is aimed at use cases in which mechanical or magnetic pulses trigger a wakeup event for an otherwise sleeping (sensor) system. In such a scheme, the efficacy of saving battery lifetime obviously depends on the duty cycle, i.e., the ratio between operating time and sleep mode.

Yamawaki et al. presented a battery life estimation calculation comparing the lifetime of a conventional wireless sensor node (WSN) with a footer transistor-based wakeup system showing that, for a moderate number of wakeup events per day (1–100), such a scheme can effectively enhance the battery life of a WSN [37]. In a similar manner, an estimated battery life comparison between a conventional periodic wakeup scheme and the proposed adaptive event-based scheme is presented in [38], where the battery life of a sensor node running in a conventional cyclic scheme is calculated by:

(2) $$L_{\mathrm{cyc}}=\frac{C\;\times \;9.7\;\times \;10^{-5}}{I_{a}D+\;I_{s}\left(1-D\right)}\;[y]\;,$$

where $L_{\mathrm{cyc}}$ is the battery life in years for the cyclic scheme, C is battery life capacity, $I_{a}$ is current during active mode, D is duty cycle, and I_S_ is current during sleep mode.

In the adaptive scheme, the sleep current I_S_ during off state is replaced by the leakage current at the transistor switch I_L_. The duty cycle D is modified to account for the number of wakeup events per day D’ = T_a_N/86400 [s]. Inserting D’ for D and I_L_ for I_S_ in Equation (2) yields for an adaptive scheme [38]:

(3) $$L_{\mathrm{adp}}=\frac{C\;\times \;8.38}{I_{a}T_{a}{N+I}_{L}{(86,400\;-\;T}_{a}N)}\;[y]\;,$$

where $L_{\mathrm{adp}}\;$is expected battery life in years, $T_{a}\;$is active period, and N is number of wakeup events per day.

In a conventional periodic wakeup scenario, the microcontroller is in one of its several low-power sleep modes. In contrast, the proposed adaptive scenario disconnects the circuit by the NMOS footer switch when not in operation. To compare the expected battery life in the adaptive scheme to the cyclic one, a simplified data acquisition and transmission cycle was analyzed for a typical WSN unit. For this purpose, the power consumption of an eZ430-RF2480 board housing an MSP430F2274 microcontroller and a CC2480 radio module was considered. Based on the datasheet for this specific device and the particular scenario, the average current consumption in active period is I_a_ = 5.15 mA and T_a_ = 66.5 ms [39]. The sleep current in the different available low-power modes of the microcontroller varies. The three low-power modes LPM0_100 kHz_, LPM2, and LPM3 were considered for comparison to the adaptive scheme, with the choice of mode depending on the application requirements. Table 2 presents a summary of the parameters and values used in Equations (2) and (3) to calculate the expected battery life.

**Figure 12:**
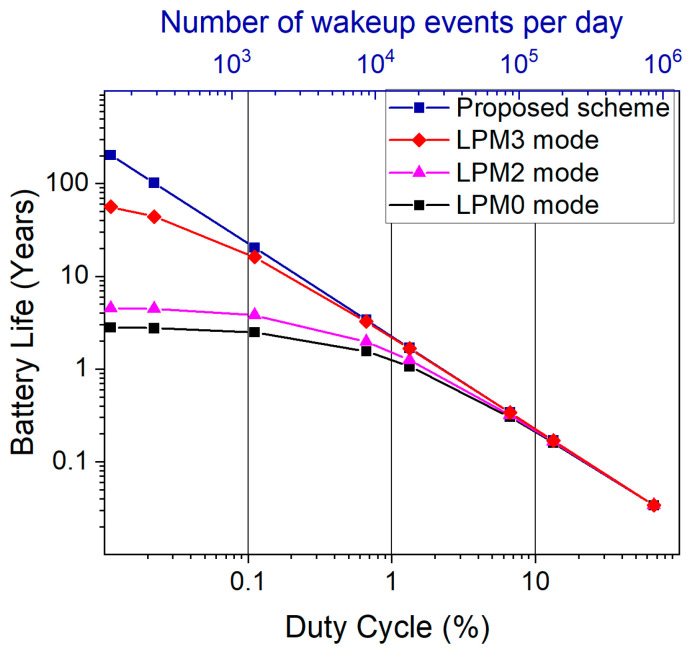
Comparison of expected battery life for cyclic and adaptive schemes with respect to corresponding duty cycle, and the number of wakeup events.

The estimation of the battery life for the different schemes and sleep modes with respect to duty cycle is depicted in Figure 12. In the investigated scenario, the proposed scheme provides an advantage over the cyclical scheme for all three low-power modes if the duty cycle is less than 10% (corresponding to data transmission every 0.67 s). When the duty cycle is kept at 0.1% (data transmission every 1 min), the adaptive scheme yields a battery life of approximately 20 years compared to 3 years in LPM0 mode. Thus, depending on the required duty cycle, an adaptive wakeup can noticeably extend the battery lifetime of a sensor.

Targeting a typical sensor lifetime of some years up to some tens of years, as investigated in Figure 12, a corresponding wakeup device must achieve a comparable operating time. The stability of similar devices (AlScN was used instead of AlN) was evaluated over the course of 150 h of continuous operation in [41]. Within this limited timeframe, the piezoelectric layer exhibited no degradation. In addition, the magnetic properties of the integrated micromagnets did not decrease during storage under ambient conditions for two years [42]. Apart from these material-based indications, a study of long-term stability on a device level needs to be conducted in future works.

## 6. Conclusions and Outlook

A contactless low-frequency zero-power wakeup was demonstrated with a MEMS energy-harvesting device featuring wafer-level-integrated permanent magnets. Wakeup times in the order of tens of ms were achieved with only few excitation pulses necessary to provide the required gate voltage in the case of excitation acceleration a_exc_ = 20–50 g and f_exc_ ≈ 40–50 Hz. The NMOS footer scheme exhibits a virtually zero leakage power loss of P_loss_ ≈ 0.1 nW resulting from the leakage current of the transistor. Thus, battery lifetime can be increased by orders of magnitude depending on the required duty cycle of a sensor node. Exploiting application-specific wakeup events, fixed wakeup intervals can be avoided which either unnecessarily consume power or miss relevant data.

Due to the flexibility of the magnetic excitation scheme, the wakeup device can be used in various scenarios such as monitoring the structural integrity of infrastructures [43,44,45], vehicle tracking [9,46], and door monitoring [37]. In addition, such wakeup devices can also be integrated where high levels of acceleration occur, e.g., when dropped objects impact from heights [47,48], and in tires of cars [49]. However, for such applications, the long-term stability of the device needs to be investigated in future work.

Based on application-specific characteristic parameters, the performance of the wakeup device can be optimized in various ways. The resonance frequency can be adapted by modification of the tip mass density (using different materials in the wafer-level integration process [20]). The electrode area and the piezoelectric volume can be varied to either achieve a further miniaturization of the MEMS device or a higher power output for faster wakeup. The number of required excitation pulses and excitation intensity (magnetic field, acceleration), as well as the wakeup time, can be improved in a future discrete or integrated solution with lower threshold voltage and higher rectification efficiency. The generated voltage output of a cantilever-based piezoelectric harvester is directly related to the piezoelectric coefficient of the material and the relative dielectric constant. With the future integration of AlScN as piezoelectric material instead of AlN, this can be expected to further increase the performance of the device by a factor of 3 to 6, due to its higher figure of merit [41,50]. This improvement will allow the device to be used at even lower frequencies, magnetic fields, and acceleration levels.

## Figures and Tables

**Table 1:** Basic design parameters of the MEMS harvesters.

Design	Tip Mass Area (Length × Width)	Beam Length	Width at Clamped End	Cantilever Volume Including Tip
H02	1.5 × 1 mm^2^	3.25 mm	3.2 mm	0.924 mm^3^
H03	1.9 × 1 mm^2^	3.05 mm	3.5 mm	1.175 mm^3^

**Table 2:** Summary of the parameters and values used for battery life estimation.

Parameter		Value [39,40]
Battery capacity, C (Ah)		1.2
Current Current consumption in active period, $I_{a}$ (mA)		5.15
Current consumption in sleep mode, $I_{s}$ (µA)	LPM0_100 kHz_	41
	LPM2	25
	LPM3	1.5
Leakage current, $I_{L}$ (pA)		31.1
Active period, $T_{a}\;$ (ms)		66.5
